# Tibial Tuberosity Fracture in an Elderly Gentleman: An Unusual Injury Pattern

**DOI:** 10.1155/2020/8650927

**Published:** 2020-03-16

**Authors:** Emma Brown, Mohammad Taaha Sohail, Jonathan West, Benjamin Davies, Georgios Mamarelis, Mohammad Zain Sohail

**Affiliations:** ^1^Foundation Year 1 Doctor, Broomfield Hospital, Mid Essex Hospital Trust, Chelmsford, Essex CM1 7ET, UK; ^2^Shifa International Hospital, H-8/4, Islamabad, Pakistan; ^3^Foundation Year 2 Doctor, University of St Andrews, St Andrews KY16 9AJ, UK; ^4^Foundation Year 2 Doctor, Broomfield Hospital, Mid Essex Hospital Trust, Chelmsford, Essex CM1 7ET, UK; ^5^Speciality Registrar in Trauma and Orthopaedics, Royal London Hospital, Whitechapel, London, UK E1 1BB; ^6^Speciality Registrar in Trauma and Orthopaedics, Colchester General Hospital, Colchester, Essex CO4 5JL, UK

## Abstract

Avulsion fracture of the tibial tuberosity is an infrequent injury in adolescents and an extremely rare occurrence in adults. We describe the case of an 86-year-old gentleman presenting after a fall, sustaining injury to the left knee. Radiographs of the left knee showed avulsion fracture of the tibial tuberosity. The purpose of this study was to present a rare case of tibial tuberosity avulsion fracture in an adult, the treatment performed, and the challenges faced. The case is discussed with the review of the literature.

## 1. Introduction

Tibial tuberosity avulsion fractures are uncommon, constituting <3% of all epiphyseal injuries and 1% of all physeal injuries in skeletally immature population [1, 2]. Such lesions are typically seen in adolescent males with well-developed quadriceps musculature and are usually incurred during jumping sports activities, such as basketball [3]. There are two possible mechanisms for this injury: strong quadriceps contraction during knee extension such as jumping or by violent flexion of the knee against a tightly contracting quadriceps, such as landing from a jump [3–5]. This type of fracture in an adult male is an extremely rare occurrence [6].

We present a case of an 86-year-old man sustaining tibial tuberosity avulsion fracture after a low energy fall. The patient underwent surgical fixation. Rehabilitation was complicated by another fall in the postoperative period leading to failure of fixation. Revision open reduction internal fixation was carried out with satisfactory results.

## 2. Case Presentation

A previously fit and well 86-year-old male presented to the emergency department after having slipped on black ice in his home garden. He reported to have fallen backwards twisting his left leg. He denied any head injury, loss of consciousness, or dizziness. He complained of a painful swollen left knee and was unable to bear weight on the left knee. The patient graded the pain as 9/10 on a visual analogue scale for pain at the time of injury.

In the patient's past medical history of note was a surgical repair of a left patella fracture. However, no detailed records of the operation or radiographs were available. There was no other significant past medical or drug history. On further questioning, the patient denied any history of previous chronic knee pain, difficulty walking, or being diagnosed with osteoporosis. The patient lives with his wife, mobilized independently without support, and is a nonsmoker.

Examination of the knee revealed a faint midline scar in the prepatellar region, extending 2 cm breadth from the superior pole of the patella to inferior pole of the patella. There was anterior knee tenderness at the tibial tuberosity, and effusion of the joint was noticed. There was no varus or valgus instability. He was unable to bear weight and unable to straight leg raise on his left side. The neurovascular status was essentially unremarkable.

**Figure 1:**
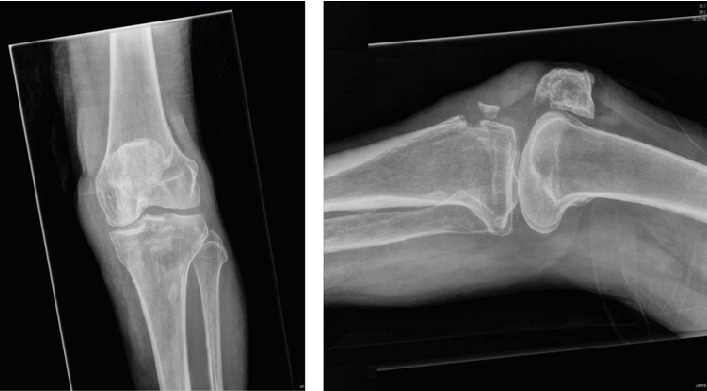
Left knee AP and lateral radiographs showing avulsion fracture of the tibial tuberosity.

Haematological investigations were all with in normal limits. Radiographs of the left knee showed an avulsion fracture of the tibial tuberosity (Figure 1).

**Figure 2:**
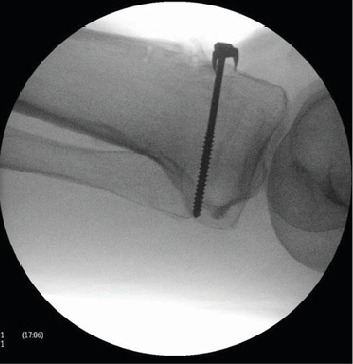
Intraoperative lateral radiograph showing fixation with spiked washer and partially threaded cancellous screws.

The patient underwent surgical fixation. Intraoperative findings showed avulsed segment of the tibial tuberosity and tears along the medial and lateral edges of patellar tendon in the extensor retinaculum. There was no striking evidence of abnormal quadriceps musculature in relation to his age. A 70 mm cancellous screw and spiked washer was passed through tendinous parts proximal to tuberosity comminution and screwed to proximal tibia. A single 5.5 mm bioabsorbable corkscrew arthrex anchor was placed into the edge of tibial tuberosity and inserted to the tibia. Extensor retinaculum tears on either side were repaired (Figure 2).

Postoperatively, the patient shows good signs of recovery. Range of motion of the knee was restricted from 0-30 degrees for the first 3 weeks using locked knee brace. The patient was discharged with 2-week follow-up appointment.

**Figure 3:**
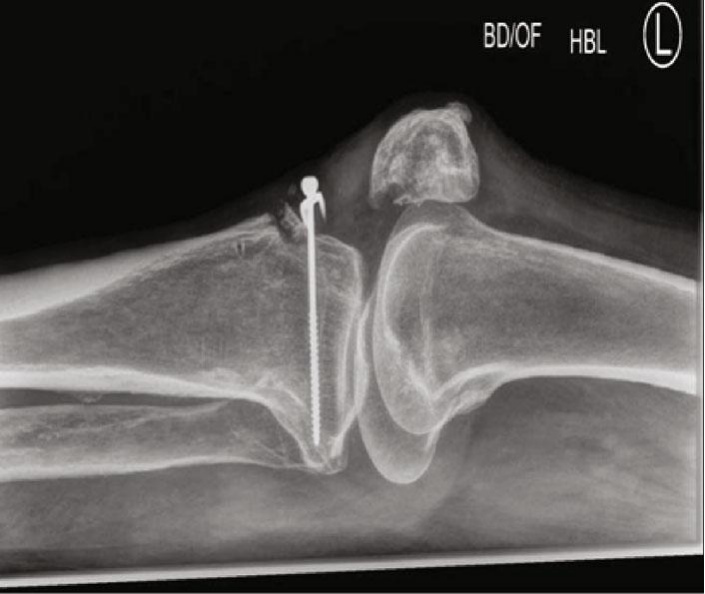
Lateral radiographs of the knee after the second injury with migration of the washer and failure of fixation.

The patient represented following a second fall and was unable to bear weight or extend his knee. The wound was healing well. Repeat radiographs of the knee showed migration of the screw and spiked washer (Figure 3). US scan of the left extensor mechanism did not reveal any abnormality. The patient underwent revision patellar tendon repair. Exploration revealed that the spiked washer and the metal work had become loose. Samples were taken for microbiology which were all essentially unremarkable. The loose bony fragments of the patellar tendon were excised, and the edges of the patellar tendon were freshened and attached to the proximal tibia using anchors and retinacular repair.

Postoperatively, his left knee remained in a cylindrical cast for 4 weeks and was then changed to a genu brace. The patient remained stable and recovered well from his revision surgery. At 8 weeks follow-up, the patient was able to walk unaided and could extend the knee with no extension lag, flexion to 90 degrees, and continued with ongoing physiotherapy.

## 3. Discussion

Tibial tuberosity avulsion fractures are an extremely uncommon presentation for adult patients. The mechanism of injury, as described above, occurs due to strong quadriceps contraction with knee extension usually the result of a jumping sports injury.

The mean age of mechanical vulnerable period for this type of injury is 14 years [7], while preadolescent cases have also been reported [8, 9]. Because male physiological physiodesis [3] leads to stronger quadriceps muscles and a greater number of males participate in athletics, males have a greater preponderance with this injury [10]. The stronger quadriceps contribute to higher traction stresses in males. Despite tibial tubercle fractures almost always occurring in males, there have been nine cases previously reported in females [7, 8, 11–14]. In the literature, there is no predominance of lesions being left or right sided as the injuries occurred with almost equal predilection, with 40 lesions being left sided and 39 being right sided out of a total of 79 [2, 5, 7, 8, 13].

The injury inadvertently occurs due to eccentric contraction of the quadriceps during pushing off or landing while jumping, resulting in severe traction on patellar tendon insertion and if the force is greater than the strength of tibial tubercle, the result will be a fracture [15]. However, Kang et al. have described the tibial tubercle fracture resulting in an 84-year-old man because of direct trauma to the knee [16]. In the case described by Kang et al., the patient was also of similar age with no previous significant medical history. He had presented with bifocal avulsion fracture of both the inferior pole of the patella and the tibial tuberosity, following a motorcycle accident where there was a higher impact between his knees and the floor.

It may occur acutely or in the presence of long standing apophysitis. Causative factors suggested include tight hamstrings, pre-existing Osgood-Schlatter disease, and disorders involving physeal abnormalities [2, 17–19], and reports of such fracture are also seen in children with osteogenesis imperfect [20]. Though an association with the Osgood-Schlatter disease has been proposed, no causal relationship has been found as suggested by Ogden and Southwick [21]. Systemic disorders such as prolonged steroid use, arthritis, chronic renal failure, and systemic lupus erythematosus have also been implied in extensor knee mechanism injuries [22]. Our patient denied previous symptomatology in bilateral knees along with any chronic systemic ailment or drug use; however, he did undergo left-sided knee surgery for fractured patella. Acute avulsion fracture of tibial tubercle has been reported in patients who underwent anterior cruciate ligament repair with autologous patellar tendon graft [10].

Modified Ogden classification of original classification provided by Sir Reginald Watson-Jones is mostly used to classify tibial tuberosity fractures, though this is described in the literature only for immature bones related to growth phase and the existing physis [19]. For this reason, no specific classification of fractures of tibial tubercle was used in our patient. However, classifying the injury based on disruption of extensor mechanism of knee was employed which is further subdivided into rupture of the quadriceps tendon, patellar fracture, patellar ligament rupture, and fracture of the tibial tuberosity [23].

X-rays of the knee are considered first-line imaging modality in confirming the diagnosis, while also a cost-effective test [3].

The management, follow-up, and complications have all been described to an exhaustive extent in the literature for immature skeletons. Numerous fixation techniques are available to address the lesions. The goals of the treatment are anatomical reduction of the displaced fragment, restoration of the extensor mechanism alignment, and maintaining the congruency of articular surface of tibia [5, 7, 24]. Open reduction of displaced bony fragments followed by fixation with cancellous screws is effective. Fracture types that extend to the joint can be managed with percutaneous cannulated screw fixation [25–27].

Fixation with various wires, suture repairs, and tension banding along with arthroscopic-assisted fixation have been previously documented in the literature with good results [25, 26].

There are opposing opinions regarding the postoperative immobilization period and whether to keep the knee immobilized or not [1]. Considering that these fractures are exceptionally rare in adults, minimally displaced, small avulsion fragments have been treated successfully [8] in adolescents by closed reduction and cast immobilization, with the knee extended for approximately 4 weeks or until the evidence of union can be seen on the radiographs [2, 7, 28]. Similarly, in severely displaced fractures, open reduction in adolescents with screws, tension band wiring, or suture repair of periosteum is done followed by casting for 3-4 weeks [29].

Complications generally include loss of flexion, non- or malunion, skin necrosis, deep venous thrombosis, preoperative compartment syndrome, and postoperative stiffness. Moreover, Blount [30] puts forward genu recurvatum as the potential complication of tibial tuberosity fracture in the young; however, there is only one case reported in an 11-year-old boy [31].

On reflection of this case, because there is no way to quantify the adequacy of the fixation during the index procedure, we believe the factors that could have led to the failure of fixation can be attributed to the second fall and/or presumed poor bone stock in elderly patients. We suggest basing the decision of adopting one of the two surgical techniques on individualized patient to patient basis, keeping in mind the bone quality. The length of immobilization followed the available literature on postoperative management of tibial tuberosity avulsion fractures. The patient underwent physiotherapy and occupational therapy throughout their admission and was discharged once able to mobilise safely. However, there is no definitive way to prevent a patient from falling; an additional step could have been to immobilise the knee for a longer period of time, as most of the literature regarding immobilization is based on children and adolescents' healing abilities.

## 4. Conclusion

In our opinion, prompt diagnosis and early management geared towards better functional outcome should be the priority in adults with acute avulsion fractures of the tibial tubercle, bearing in mind that this lesion should be treated as a separate entity in elderly patients with attentive details given to bone stock and the duration of immobilization.

Further research could be carried out for the aetiology, incidence and treatment options, and prolonged patient reported outcomes for tibial tubercle fractures in geriatric age group.
